# Carnosine and Acyl Carnitines as Metabolic Determinants of Muscle Phenotypic Differences Between Longissimus Dorsi and Triceps Brachii in Hanzhong Sheep

**DOI:** 10.3390/foods14193289

**Published:** 2025-09-23

**Authors:** Zhi Li, Miaohua Zheng, Weiwei Li, Jiayi Li, Ling Wang, Shanshan Wang, Hongzhao Lu, Tao Zhang

**Affiliations:** 1 School of Biological Science and Engineering, Shaanxi University of Technology, Hanzhong 723001, China; 17691209412@163.com (Z.L.); 15929301569@163.com (M.Z.); 13488386146@163.com (W.L.); 15596158097@163.com (J.L.); wangling619@163.com (L.W.); jieyishanshan319@163.com (S.W.); 2 Shaanxi University Engineering Research Center of Quality Improvement and Safety Control of Qinba Special Meat Products, Hanzhong 723001, China; 3 Shaanxi Union Research Center of University, and Enterprise for Zhenba Bacon, Hanzhong 723001, China; 4 Qinba State Key Laboratory of Biological Resources and Ecological Environment, Hanzhong 723001, China

**Keywords:** Hanzhong sheep, longissimus dorsi muscle, triceps brachii muscle, meat quality differences, carnosine, acylcarnitine

## Abstract

Muscle traits are critical determinants of meat quality and productivity in sheep, influenced by both breed and anatomical region. Hanzhong sheep, an indigenous Chinese breed, are prized for tender, low-odor meat; yet, the molecular mechanisms underlying these traits remain poorly understood. In this study, we integrated meat quality assessment with metabolomic and transcriptomic profiling of the longissimus dorsi (HZ-B) and triceps brachii (HZ-T) muscles to elucidate biochemical and molecular bases of regional differences. The results, derived from metabolomic profiling, demonstrated that the muscle tissue of Hanzhong sheep contained abundant proteins (95 kinds) and fatty acids (150 kinds). The greater tenderness of HZ-B compared to HZ-T was associated with higher levels of dipeptides such as carnosine (FC = 1.07) and anserine (FC = 1.04), as well as upregulated expression of oxidative fiber-related genes MYH2 (FC = 2.92) and TPM1 (FC = 2.15). In contrast, HZ-T showed enrichment of flavor-associated metabolites, including acylcarnitines and glutamate, alongside higher expression of *FBXO32* (FC = 0.35) and *MYBPC1* (FC = 0.47), linked to structural integrity and muscle contraction. Integrated analysis revealed strong associations between metabolite abundance (carnosine/anserine) and key genes (*FBXO32*/*GADL1*), suggesting coordinated regulation of meat quality traits. These findings provide mechanistic insights into the metabolic and transcriptomic determinants of muscle quality in Hanzhong sheep, offering a foundation for genetic improvement and conservation strategies.

## 1. Introduction

Lamb meat is an important component of global meat consumption due to its high protein content, relatively low fat levels, and distinctive flavor profile. These quality attributes directly influence culinary performance, sensory characteristics, processing suitability, and marketability throughout commercial supply chains [1]. Meat quality in livestock is affected by multiple factors, with intra-species variation largely attributable to differences in rearing environments, post-mortem treatments, and anatomical regions. Such regional heterogeneity is recognized as a fundamental biological phenomenon, as reflected by substantial divergence in muscle fiber composition and metabolic characteristics across distinct anatomical sites in sheep and other livestock [2,3]. Contemporary studies have demonstrated that muscle fiber typology, structural properties, and intramuscular fat deposition are key determinants of regional variation in meat quality [4,5]. The influence of anatomical location is well-established in ovine research. Anderson et al. [6] reported region-specific patterns of intramuscular fat deposition in different lamb carcass parts. Lu Dou et al. [7] reported significant differences in malondialdehyde content, antioxidant activity, and volatile flavor compounds between the quadriceps femoris and longissimus dorsi muscles of lambs.

In recent years, integrated multi-omics approaches have emerged as powerful tools in life science research, providing new insights into the biochemical basis of complex traits and enabling the identification of diagnostic and prognostic biomarkers. In meat science, these approaches have been widely applied to investigate muscle physiology and meat quality [8]. Transcriptomics allows for the systematic characterization of gene expression profiles in muscle tissues, whereas untargeted metabolomics provides a comprehensive overview of metabolite diversity. Integrating these datasets enables the identification of pivotal biomarkers and the construction of regulatory interaction networks [9,10]. For instance, Qian et al. [11] demonstrated through transcriptomic analysis that the PI3K/AKT signaling pathway plays a critical role in maintaining metabolic homeostasis in beach lamb during post-mortem storage, thereby supporting muscle development. Zhang et al. [12] applied integrated transcriptomic and metabolomic analyses to compare meat quality traits between Hu sheep and F1 hybrids with Nanqiu sheep, revealing that crossbreeding significantly modulates key signaling pathways and gene expression. Han et al. [13] further showed that transcriptomic-metabolomic profiling can uncover significant effects of gender and age on the chemical composition and flavor characteristics of Huishan goat meat.

Hanzhong sheep, a short, thin-tailed breed representing an ancient indigenous lineage, is well recognized for its tender meat, uniform fat distribution, and bright muscle coloration. Previous studies on this breed have largely focused on resource surveys, conservation genetics, and nutritional management. Although its desirable meat quality traits—such as fine texture and rich flavor—have been well documented [14], the molecular mechanisms underlying these traits remain poorly understood. In particular, the role of part-specific differences in shaping meat quality has not yet been elucidated using multi-omics strategies. Therefore, comprehensive characterization of the fundamental meat quality attributes of Hanzhong sheep and deciphering the molecular basis of region-specific variation through integrated omics analyses are essential. Such efforts will provide a foundation for genetic resource conservation, breeding improvement, meat quality benchmarking, and the sustainable utilization of this heritage breed.

## 2. Materials and Methods

### 2.1. Animals and Muscle Samples

Thirty healthy male Hanzhong sheep were reared under standardized environmental and nutritional conditions until seven months of age. From this cohort, six animals with comparable body weights were randomly selected for slaughter and subsequent sample collection. Prior to slaughter, sheep were subjected to a 24 h fasting period, with water withheld during the final 12 h. Animals were transported to a commercial slaughterhouse and humanely euthanized in accordance with established welfare guidelines, specifically by stunning followed by exsanguination to ensure rapid loss of consciousness and death without distress.

The longissimus dorsi (HZ-B), a stabilizing muscle primarily composed of glycolytic fibers and known for its tenderness, and the triceps brachii (HZ-T), a locomotor muscle with distinct fiber composition and flavor characteristics [15,16], were sampled from each carcass within 30 min postmortem (*n* = 6). Tissues were trimmed of visible connective tissue and divided into aliquots for downstream analyses. Histological samples were immersion-fixed in 4% paraformaldehyde (PFA, pH 7.4) at 4 °C. Samples designated for biochemical assays were stored at −20 °C, while those for omics analyses were snap-frozen in liquid nitrogen and stored at −80 °C in an ultra-low temperature freezer.

### 2.2. Slaughter Performance and Meat Quality Assessment

Pre-slaughter live weight and body size indices (body length, height, cannon circumference, and cross-sectional width) were recorded with animals in an upright posture. Slaughter procedures were conducted in accordance with the Standards for Livestock and Poultry Slaughter (GB/T 35892-2018) [17], including sequential removal of the head, hooves, skin, hair, and viscera (excluding kidneys). Carcasses were trimmed, subjected to lymphadenectomy, rested for 30 min, and weighed. Backfat thickness and GR values were measured at the mid-dorsal spine and between the 12th and 13th ribs, respectively.

Muscle pH values were determined at 45 min and 24 h postmortem by inserting a portable pH meter probe at a depth of 2 cm into both the longissimus dorsi and triceps brachii. Physicochemical indices were assessed within 24 h postmortem. Shear force was measured by heating samples in a water bath at 80 °C until an internal temperature of 70 °C was reached, followed by analysis with a shear force meter. Cooking loss was calculated as the percentage reduction in weight after cooking samples in a water bath at 85 °C for 20 min. Drip loss was determined as the percentage of weight loss after suspending samples at 4 °C for 24 h [18].

For texture profile analysis (TPA), samples were cut into cubes (3 cm × 3 cm × 1 cm) and tested using a Brookfield CT3 texture analyzer with a cylindrical TA5 probe (5 mm diameter). Instrument calibration was performed according to the manufacturer’s specifications. Parameters were set as follows: pre-test speed 1.0 mm/s, test speed 1.0 mm/s, target strain 50%, and two compression cycles. Five indices were recorded: hardness, elasticity, cohesiveness, chewiness, and resilience.

### 2.3. Hematoxylin-Eosin (HE) Staining of Muscle Tissue

Paraformaldehyde-fixed muscle samples were rinsed under running water for 30 min, dehydrated in graded ethanol, cleared in xylene, and paraffin-embedded. Sections (3–6 µm) were cut, dewaxed, and rehydrated. Staining was performed with hematoxylin for 3 min, differentiation solution treatment, and eosin staining for 3 min, followed by dehydration, clearing, and sealing with neutral resin. Muscle fiber morphology was observed under a light microscope. The diameters, cross-sectional areas, and densities of 45 randomly selected fibers per section were quantified using Image Pro Plus (v6.0).

### 2.4. Metabolite Extraction and LC-MS Analysis

**Figure A1:**
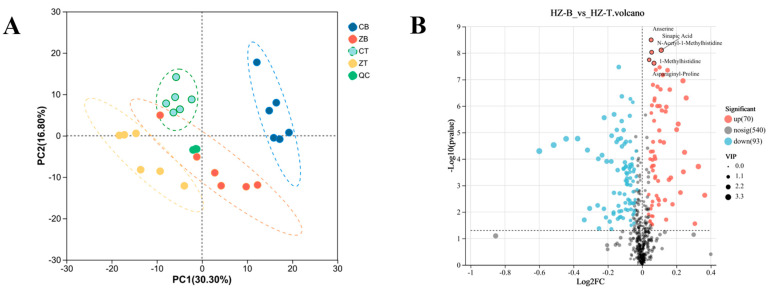
Muscle tissue PCA and volcano plot: (**A**) PCA of metabolic products of the longissimus dorsi and triceps brachii muscles of Hanzhong sheep and their F1 hybrid with Suffolk sheep, and (**B**) differential metabolite volcano plots of the longissimus dorsi and triceps brachii muscles.

Approximately 50 mg of muscle tissue was homogenized in a 2 mL centrifuge tube containing 6 mm grinding beads and 400 µL extraction solution. Quality control (QC) samples were pre-pared by pooling aliquots of all samples processed within the same experimental batch; this included the Hanzhong sheep samples analyzed in this study, in addition to samples from a parallel investigation on crossbred sheep (Figure A1). This pooled QC sample was employed to monitor instrumental performance and correct for technical variation during data acquisition. Metabolomic profiling was performed on a UHPLC-Q Exactive HF-X system (Thermo Fisher Company, Waltham, MA, USA). Chromatographic separation was achieved on a C18 column (2.1 × 100 mm, 1.7 µm) at 40 °C, with a flow rate of 0.3 mL/min. The original data were peak aligned, baseline corrected, and matched to databases such as HMDB (versions 5.0), Metlin (Version 1.0.0r00), and in-house libraries using Progenesis QI software (Waters Corporation, Milford, USA). Multivariate analyses, including principal component analysis (PCA) and orthogonal partial least squares discriminant analysis (OPLS-DA) models were constructed using the ropls package in R (Version 1.6.2). Differential metabolites were identified with thresholds of VIP > 1 and *p* < 0.05. Pathway enrichment analysis was performed using Fisher’s exact test based on KEGG (kegg_v20230830) pathway annotation combined with SciPy.stats (Version1.0.0). The resulting data were analyzed and visualized using the online cloud platform at cloud.majorbio.com [19,20].

### 2.5. Total RNA Extraction and Transcriptomic Analysis

Total RNA was extracted from muscle tissue using a TRIzol-based reagent kit (QIAzolLysisReagent, Qiagen, Hilden, Germany). RNA concentration and purity were assessed using a NanoDrop 2000 (Thermo Fisher Scientific, Waltham, MA, USA) spectrophotometer, while RNA integrity was evaluated with an Agilent 5300 Bi-oanalyzer system (Agilent, Santa Clara, CA, USA). Only samples with an RNA integrity number (RIN) ≥ 6.5 were utilized for subsequent library construction. To ensure the robustness and reliability of the statistical models against potential batch effects, a rigorous permutation test (200 iterations) was performed on the OPLS-DA models. Transcriptome libraries were constructed using the TruSeq Stranded Total RNA Li-brary Prep Kit (Clontech, Mountain View, CA, USA), following the manufacturer’s protocol. Paired-end sequencing (150 bp) was performed on an Illumina NovaSeq 6000 platform (Illumina, San Diego, CA, USA). Raw sequencing reads underwent quality control and adapter trimming using FastQC and Trimmo-matic, respectively, and were subsequently aligned to the sheep reference genome (GCF_002742125.1) using the STAR aligner. Gene and transcript abundance was quantified using RSEM (v1.3.3). Differential gene expression analysis was performed with the DESeq2 package (v1.42.0), with genes satisfying the thresholds of an adjusted *p*-value (FDR) < 0.05 and |log2(fold change)| ≥ 1 considered significantly differentially expressed genes (DEGs). Functional enrichment analysis of Gene Ontology (GO, Version 2023.07) terms and Kyoto Encyclopedia of Genes and Genomes (KEGG, Version 2023.09) pathways was conducted based on the identified differentially expressed genes using GOA Tools (https://github.com/tanghaibao/GOatools (accessed on 19 September 2025)) and KOBAS software (https://www.kobas.co.uk/), respectively, with statistical significance determined by Fisher’s exact test. All data analyses were performed on the Meiji Online cloud computing platform [21,22].

### 2.6. Comprehensive Analysis of Transcriptome and Metabolome Data

Pearson correlation analysis was used to evaluate associations between DEGs and differential metabolites (DMs). Correlation heatmaps were generated, and network visualizations were constructed in Cytoscape (Version 3.10.0) with significance set at *p* < 0.05. KEGG-based co-enrichment analyses of DEGs and DMs were performed, and significantly enriched pathways were identified (*p* < 0.05).

### 2.7. Validation of DEGs via Real-Time Quantitative PCR

Seven DEGs were selected for validation using RT-qPCR, with GAPDH as the reference gene. Each gene was analyzed in triplicate. Reactions were conducted as follows: initial denaturation at 95 °C for 30 s; amplification at 95 °C for 5 s and 60 °C for 5 s (40 cycles); final extension at 95 °C for 15 s, 60 °C for 1 min, and 95 °C for 15 s. Relative expression levels were calculated using the 2^−ΔΔCt^ method and statistically analyzed in GraphPad Prism 9. The detailed information of the primers can be found in Appendix A.

### 2.8. Statistics and Analysis of Data

Data were presented as mean ± standard deviation (SD). Statistical differences between groups were assessed using one-way analysis of variance (ANOVA), followed by appropriate post hoc tests when necessary. A significance threshold of *p* < 0.05 was applied for all analyses. Graphical representation and statistical processing were conducted using GraphPad Prism 9 (Version 9.5.0, USA).

## 3. Results

### 3.1. Hanzhong Sheep Exhibit Small Body Size, Slow Growth and Superior Tenderness

**Figure 1:**
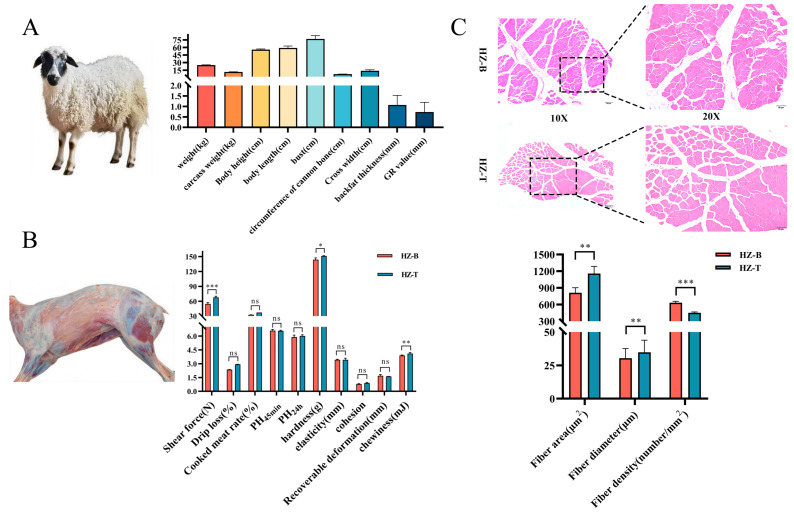
Growth performance and meat physicochemical characteristics of Hanzhong sheep. (**A**) Body size indices; (**B**) comparison of meat quality parameters between HZ-B and HZ-T; and (**C**) muscle fiber morphological characteristics (values are mean ± SD, *n* = 6; * 0.01 < *p* ≤ 0.05, ** 0.001 < *p* ≤ 0.01, *** *p* ≤ 0.001); “ns” denotes not statistically significant.

Hanzhong sheep in this study presented a small body conformation, with a body height of 55.67 ± 1.53 cm, body length of 59.00 ± 4.00 cm, chest circumference of 77.00 ± 6.56 cm, cross-sectional width of 7.10 ± 0.17 cm, and cannon bone circumference of 13.67 ± 2.09 cm. The mean live body weight was 25.07 ± 0.50 kg and the mean carcass weight was 11.34 ± 0.45 kg, corresponding to a slaughter rate of 45.23%. Backfat thickness and GR value were 1.07 ± 0.46 mm and 0.74 ± 0.46 mm, respectively (Figure 1A). Comparison of meat physical properties revealed clear intermuscular differences. The longissimus dorsi (HZ-B) showed significantly lower shear force (54.75 ± 2.64 N), hardness (143.83 ± 3.72 g), and chewiness (3.89 ± 0.05 mJ) than the triceps brachii (HZ-T: 67.91 ± 2.21 N, 151.40 ± 0.58 g, and 4.10 ± 0.11 mJ, respectively) (*p* < 0.05), indicating superior tenderness and a more favorable texture profile in HZ-B. No significant differences were detected between the two muscles for cooking loss, pH, springiness, or cohesiveness (*p* > 0.05) (Figure 1B).

Histomorphometric analysis demonstrated marked regional variation in muscle fiber architecture. The longissimus dorsi exhibited a 29.81% smaller mean fiber cross-sectional area and a 12.58% smaller mean fiber diameter compared with the triceps brachii (*p* < 0.01), alongside a 29.02% higher fiber density (*p* < 0.001) (Figure 1C). Collectively, these data indicate substantial intermuscular heterogeneity in Hanzhong sheep, with the longissimus dorsi characterized by finer fiber morphology and greater tenderness relative to the triceps brachii.

### 3.2. Muscle Metabolome of Hanzhong Sheep Is Compositionally Rich and Regionally Distinct

**Figure 2:**
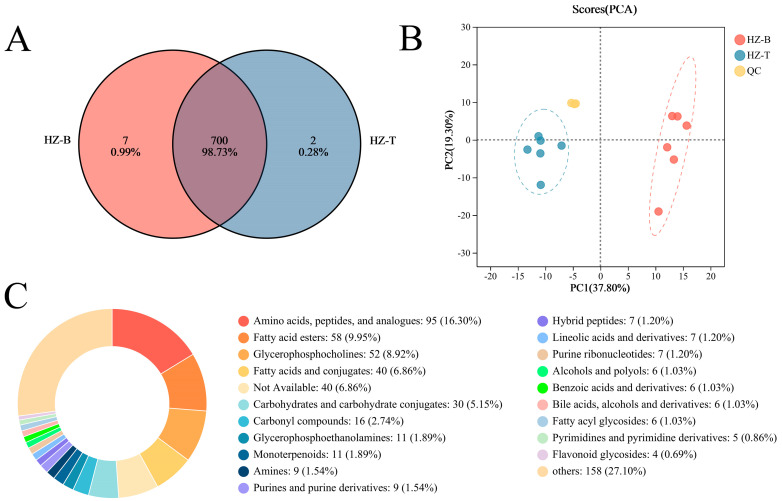
Overview of muscle metabolite characteristics in Hanzhong sheep. (**A**) Venn diagram of metabolites detected in HZ-B and HZ-T; (**B**) PCA score plot of HZ-B, HZ-T and QC samples; (**C**) HMDB classification and relative composition of detected metabolites.

Untargeted metabolomic profiling detected a total of 709 metabolites across the longissimus dorsi and triceps brachii samples: 707 metabolites were found in HZ-B and 702 in HZ-T, with 700 metabolites shared by both muscle types (Figure 2A). Although the two muscles shared broadly similar metabolite categories, their relative abundances differed substantially. Principal component analysis (PCA) revealed a clear separation between the metabolomic profiles of HZ-B and HZ-T (Figure 2B), indicating distinct metabolic signatures between these anatomical regions; QC samples clustered tightly, supporting the stability and reproducibility of the dataset.

Shared metabolites were classified into 21 categories (20 of which were annotated). The largest class was amino acids, peptides and their analogs (95 species, 16.3%), followed by fatty acid esters (58 species, 9.95%) and glycerophospholipids (52 species, 8.92%). Other represented classes included fatty acids and conjugates (40 species), carbohydrates (30 species), carbonyl compounds, glycerophosphorylethanolamines, monoterpenes, amines, purines and derivatives, heterotrimeric peptides, linoleic acid derivatives, and a range of other metabolites (totaling 158 species) (Figure 2C). The prevalence of amino acids, lipid species and flavor-related compounds in the metabolome was consistent with the previously observed favorable organoleptic properties of Hanzhong sheep meat.

### 3.3. Significant Differences in Metabolite Content Between the Longissimus Dorsi and Triceps Brachii Muscles

**Figure 3:**
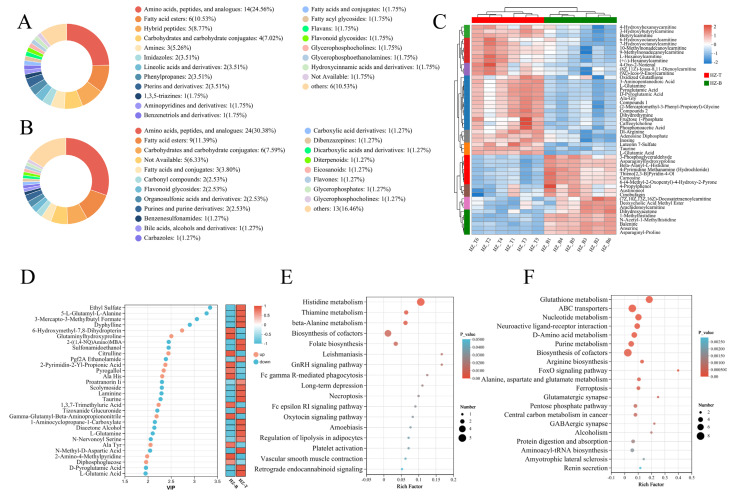
DMs profiling and pathway enrichment analysis of HZ-B and HZ-T groups: (**A**) HMDB classification of up-regulated DMs, (**B**) HMDB classification of down-regulated DMs, (**C**) heatmap of the top 50 DMs between HZ-B and HZ-T, (**D**) VIP plot of DMs, and (**E**,**F**) KEGG pathway enrichment of upregulated (**F**) and downregulated DMs.

Significant intermuscular variation in metabolite composition was observed between the longissimus dorsi and triceps brachii muscles of Hanzhong sheep. Specifically, seven metabolites were uniquely detected in the longissimus dorsi muscle—1,3,7-trimethyluric acid, PC(PGJ2/17:0), D-fructose-1,6-bisphosphate, pyrogallol, Ala-Leu, ozolinone, and diphosphoglucose—whereas two metabolites, Pro-Val and isodeoxycholic acid, were uniquely present in the triceps muscle (Figure 3A). Several of these metabolites are functionally linked to meat quality. For example, 1,3,7-trimethyluric acid, a major caffeine metabolite, and pyrrolidine glucoside, a polyphenol derivative, possess antioxidant activity that may delay lipid oxidation during postmortem aging, thereby enhancing meat preservation. In addition, the accumulation of fructose-1,6-bisphosphate, a key glycolytic intermediate, in the longissimus dorsi may contribute to its superior tenderness.

**Figure A2:**
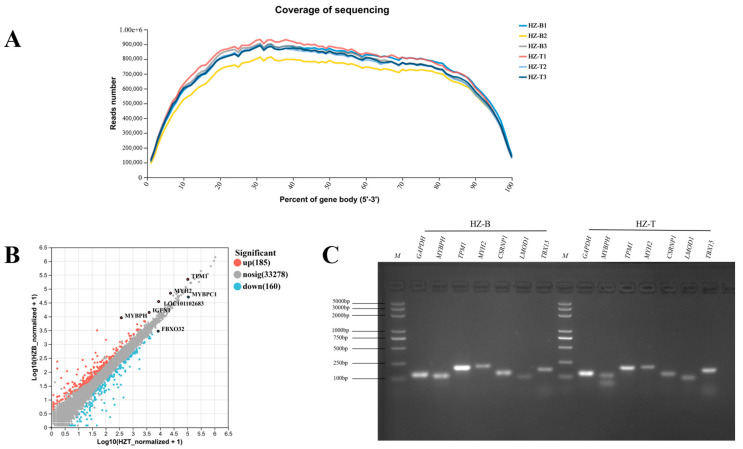
The quality control of the transcriptomes of the longissimus dorsi and triceps brachii muscles of Hanzhong sheep, the scatter plot of differentially expressed genes, and the agarose gel electrophoresis patterns of qPCR primers. (**A**), the horizontal axis represents the percentage of the base length of a single gene relative to the total base length, with 0 indicating the 5′ end of the gene and 100 indicating the 3′ end of the gene; the vertical axis represents the total number of sequence segments within the corresponding intervals on the horizontal axis that are aligned to all genes, (**B**) Scatter plot of differentially expressed genes, with the *x*-axis and *y*-axis representing the expression levels of genes/transcripts in the two samples respectively, and (**C**) The agarose gel electrophoresis pattern of 2% concentration qPCR primers: Using TAE electrophoresis buffer, with a voltage of 85 V, and the electrophoresis duration of 45 min.

A comparative metabolomic analysis identified 163 differential metabolites (DMs) between the two muscle types, with 70 upregulated in the longissimus dorsi and 93 upregulated in the triceps brachii (using the triceps muscle as the reference; Figure A2). To visually compare the differential composition of up- and down-regulated metabolites between muscle types, we separately categorized them based on HMDB classification, as shown in Figure 3A,B. These metabolites were classified into 19 categories, with six predominant groups shared between muscles: amino acids/peptides/analogs, fatty acid esters, carbohydrates, fatty acid conjugates, flavonoid glycosides, and glycerophospholipids. Among these, amino acids/peptides/analogs and fatty acid esters were most abundant, each contributing more than 10% to the total metabolite pool. Notably, the longissimus dorsi exhibited significantly higher levels of heteropeptides (e.g., carnosine and anserine), amines, and imidazole derivatives, while the triceps brachii contained elevated levels of carbonyl compounds, organic sulfonic acids and derivatives, and purines (Figure 3A,B). These differences likely underlie the distinct tenderness of the longissimus dorsi and the unique flavor attributes of the triceps brachii.

Cluster analysis of the top 50 DMs further supported these findings (Figure 3C). The longissimus dorsi was enriched in amino acids and heteropeptides such as 1-methylhistidine, carnosine, and anserine, as well as glucose metabolism-related products including glycerol-3-phosphate and dihydroxyacetone. Conversely, the triceps brachii contained significantly higher levels of flavor-related compounds, such as acylcarnitines, L-glutamine, L-glutamic acid, inosinic acid, oxidized glutathione, and taurine. These results suggest that the biochemical basis of meat quality divergence lies in the tenderness-associated metabolites of the longissimus dorsi and the flavor-related metabolites of the triceps brachii. Variable importance in projection (VIP) analysis further highlighted this divergence, showing higher levels of citrulline and L-alanyl-L-histidine in the longissimus dorsi, whereas L-glutamine, L-glutamic acid, carnosine, and taurine were more abundant in the triceps brachii (Figure 3D).

To further elucidate the biochemical pathways underlying these metabolite differences, KEGG enrichment analysis was performed (Figure 3E,F). In the longissimus dorsi, differential metabolites were significantly enriched in pathways related to histidine metabolism, thiamine metabolism, β-alanine metabolism, oxytocin signaling, and adipocyte lipolysis, which are primarily associated with muscle structure stabilization and energy balance. In contrast, the triceps brachii exhibited enrichment in pathways including glutathione metabolism, ABC (ATP-binding cassette) transporters, purine metabolism, D-amino acid metabolism, FoxO signaling, alanine/aspartate/glutamate metabolism, the pentose phosphate pathway, and amyotrophic lateral sclerosis-related pathways, which are closely linked to the accumulation of flavor precursors. Collectively, these results demonstrate that the longissimus dorsi muscle is metabolically predisposed to enhanced tenderness and structural stability, whereas the triceps brachii is enriched in pathways supporting flavor development.

### 3.4. Transcriptomic Profiling Reveals Gene Expression Differences Between Muscles

**Figure 4:**
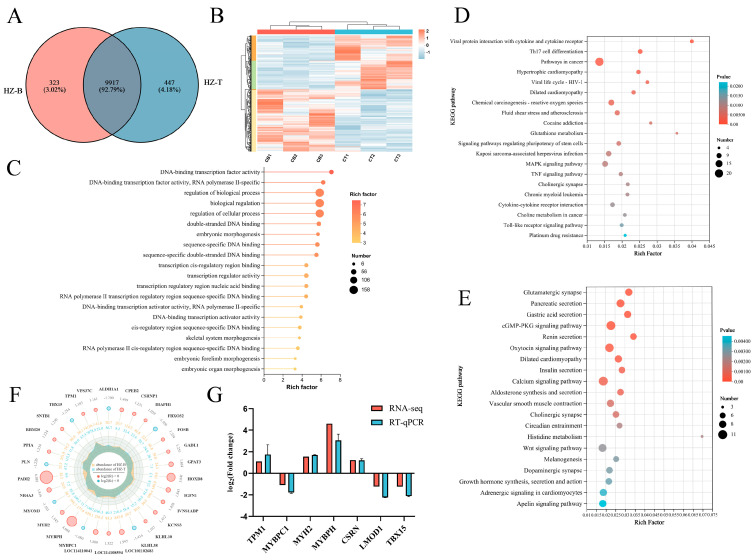
Differential gene expression profiles between HZ-B and HZ-T groups: (**A**) Venn diagram of shared and unique expressed genes between HZ-B and HZ-T, (**B**) heatmap of DEGs showing intra-group consistency and inter-group separation, (**C**) multi-dimensional GO-enriched circle plot of DEGs, (**D**,**E**) KEGG pathway enrichment of DEGs: (**D**) upregulated in HZ-B; (**E**) upregulated in HZ-T, (**F**) radar plot of the top 30 DEGs between HZ-B and HZ-T, and (**G**) RT-qPCR validation of representative DEGs.

Gene expression patterns are strongly associated with livestock growth and development, and distinct transcriptional profiles are often observed across different anatomical regions. To investigate the molecular mechanisms underlying the differences between the longissimus dorsi (HZ-B) and triceps brachii (HZ-T) muscles in Hanzhong sheep, transcriptome sequencing was performed following non-targeted metabolomic profiling. A total of 9917 genes were commonly expressed in both muscles, with 323 genes uniquely expressed in HZ-B and 447 genes uniquely expressed in HZ-T (Figure 4A). Using HZ-T as the reference, 185 genes were significantly upregulated and 160 genes were significantly downregulated in HZ-B (Figure A2). Cluster analysis of differentially expressed genes (DEGs) revealed strong intra-group consistency and clear inter-group separation, highlighting substantial transcriptional divergence between the two muscle types (Figure 4B).

Functional enrichment analyses were conducted to further explore the biological roles of DEGs. Gene Ontology (GO) enrichment showed that DEGs were mainly involved in the Biological Process (BP) and Molecular Function (MF) categories, including DNA-binding transcription factor activity, regulation of biological processes, RNA polymerase II-specific transcription, and embryonic forelimb morphogenesis (Figure 4C). KEGG pathway analysis demonstrated distinct functional enrichment between the two muscle regions. Upregulated DEGs in HZ-B were enriched in pathways related to amino acid metabolism and oxidative stress regulation, including Th17 cell differentiation, glutathione metabolism, MAPK signaling, TNF signaling, β-alanine metabolism, and adipocyte lipolysis (Figure 4D). Conversely, upregulated DEGs in HZ-T were enriched in pathways associated with muscle growth and development, such as the cGMP–PKG signaling pathway, Wnt signaling, histidine metabolism, growth hormone synthesis and secretion, and the oxytocin signaling pathway (Figure 4E). These findings suggest that the longissimus dorsi muscle emphasizes homeostasis and metabolic regulation, whereas the triceps brachii muscle prioritizes growth and structural development.

To identify key candidate genes, the top 30 DEGs were screened according to expression levels and statistical significance. In HZ-B, significant upregulation was observed for *MYBPH*, *MYH2*, *TPM1*, and *IGFN1*, which are involved in myofiber proliferation and differentiation, as well as *GADL1*, a gene related to carnosine synthesis. In contrast, HZ-T showed higher expression of *MYBPC1*, *MYOM3*, *ALDH1A1*, and *TBX15*, which maintain myofiber structural integrity, along with atrophy-related genes *FBXO32* and *KLHL38* (Figure 4F). Validation by RT-qPCR confirmed consistency with transcriptomic results (Figure 4G). Collectively, these transcriptomic findings indicate that distinct sets of genes regulating myofiber development, differentiation, and structural stability contribute to the observed differences in muscle composition and meat quality between anatomical regions in Hanzhong sheep.

### 3.5. Correlation Between DEGs and DMs in Longissimus Dorsi and Triceps Brachii Muscles

Gene expression patterns and metabolite abundances jointly underpin phenotypic traits. Previous studies in Hanzhong sheep have demonstrated marked transcriptomic–metabolomic divergence across distinct anatomical muscle regions. To elucidate the molecular basis of phenotypic differences between the longissimus dorsi and triceps brachii, we integrated transcriptomic (345 DEGs) and metabolomic (168 DMs) datasets using Spearman correlation analysis. The analysis focused on the top 30 most abundant DMs and the top 25 most highly expressed DEGs.

**Figure 5:**
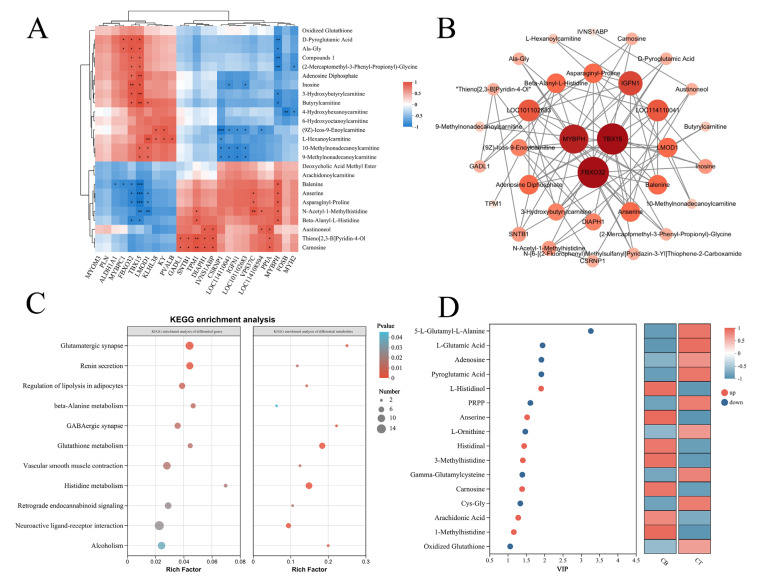
Integrated metabolomic and transcriptomic analyses of longissimus dorsi and triceps brachii muscles. (**A**) Heatmap of Spearman correlations between DEGs and DMs (* 0.01 < *p* ≤ 0.05, ** 0.001 < *p* ≤ 0.01, *** *p* ≤ 0.001), (**B**) correlation network illustrating key regulatory genes and metabolites, (**C**) KEGG pathway enrichment analysis of DEGs and DMs, and (**D**) VIP diagram of DMs in amino acid metabolism and lipid metabolism-related pathways.

In the longissimus dorsi, highly expressed structural and regulatory genes, including *MYBPH*, *TPM1*, and *GADL1*, exhibited significant positive correlations (*p* < 0.05) with antioxidant dipeptides such as carnosine and anserine. Conversely, in the triceps brachii, *MYBPC1*, *TBX15*, and *FBXO32* were negatively correlated with carnosine analogs, but positively correlated with metabolites involved in energy and lipid metabolism, including inosine, carnitines, and glutamate (Figure 5A). Correlation network topology analysis further identified *TBX15*, *FBXO32*, and *MYBPH*, together with key metabolites such as β-alanyl-L-histidine (carnosine) and anserine, as central regulatory nodes, highlighting their roles in muscle function and meat quality (Figure 5B).

Joint KEGG pathway enrichment analysis of DEGs and DMs revealed that lipolysis regulation, β-alanine metabolism, histidine metabolism, and glutathione metabolism were significantly enriched in both muscle types (Figure 5C). Within these pathways, histidine, 3-methylhistidine, carnosine, and arachidonic acid were significantly more abundant in the longissimus dorsi, whereas glutamate, pyroglutamate, adenosine, and oxidized glutathione were significantly less abundant compared with the triceps brachii (Figure 5D). These results suggest that muscle-specific expression of structural and atrophy-related genes, together with differential enrichment of antioxidant dipeptides and amino acid metabolites, constitute key determinants of meat quality variation between muscle regions.

## 4. Discussion

Meat quality in sheep is influenced by a range of intrinsic and extrinsic factors, including breed, anatomical region, sex, and age. Hanzhong sheep are locally prized for their tender texture, uniform fat distribution, and bright muscle color [14]. However, their relatively slow growth rate and small frame size impose substantial economic limitations. For instance, in this study the average body weight of 7-month-old Hanzhong sheep was only 25.07 ± 0.50 kg, markedly lower than the 53.32 ± 4.32 kg reported for 6-month-old Hu sheep [23]. This discrepancy underscores the suboptimal production performance of Hanzhong sheep, which may be linked to inherent genetic characteristics, husbandry practices, or environmental factors. Addressing these limitations requires a clearer understanding of the molecular and metabolic mechanisms governing muscle development and meat quality, thereby informing strategies for genetic improvement.

The metabolic profile of Hanzhong sheep muscle is characterized by a high abundance of nitrogenous and lipid compounds, which is consistent with its high nutritional value. The overall metabolite composition predominantly comprised amino acids, peptides, fatty acids, and glycerophospholipids. Furthermore, the metabolites that were differentially abundant between muscle types were primarily associated with amino acid and peptide derivatives, in addition to various lipid-related compounds. These differential metabolites may constitute the material basis underlying the quality differences between the longissimus dorsi and triceps brachii muscles.

Muscle fiber structure plays a pivotal role in tenderness, a critical determinant of consumer acceptability. Reduced fiber diameter and increased fiber density are strongly associated with lower shear force and enhanced tenderness [24]. In this study, the longissimus dorsi (HZ-B group) displayed superior muscle fiber characteristics compared with the triceps brachii (HZ-T group), consistent with previous findings on anatomical variation in meat quality [25]. Oxidative fibers (Type I/IIa) are generally linked with improved pH stability, lower shear force [26], and enhanced water-holding capacity [27,28]. Certain imidazole dipeptides, notably carnosine and anserine, further contribute to tenderness through their roles in pH buffering, antioxidant defense [29], and muscle differentiation [30,31]. Dietary modulation of carnosine signaling has been shown to increase oxidative fiber composition and improve structural properties [31]. In line with this, the enrichment of carnosine and anserine in the longissimus dorsi was accompanied by reduced shear force and enhanced oxidative gene expression (e.g., *MYH2*, *TPM1*), suggesting a mechanistic link whereby these metabolites promote oxidative fiber development and mitigate structural resistance.

Flavor is another central attribute of meat quality, with mutton odor being a major barrier to consumer acceptance. This odor largely originates from branched-chain fatty acids (e.g., MOA, EOA) [32], while lipid metabolism contributes flavor precursors that shape meat aroma [33,34]. Interestingly, no odor-associated branched-chain fatty acids were detected in either muscle type. Instead, the triceps brachii exhibited significant enrichment of carnitine derivatives, including isovaleryl-L-carnitine and 2-methylbutyrylcarnitine, suggesting enhanced carnitine-mediated β-oxidation and lipid remodeling [35]. Such pathways may expand the precursor pool for flavor development. Concurrently, elevated glutamate and oxidized glutathione (GSSG) in the triceps brachii are consistent with the enhancement of umami flavor [36]. Conversely, the enrichment of carnosine in the longissimus dorsi may modulate flavor indirectly by preventing lipid peroxidation and aldehyde formation through metal ion chelation [37]. Transcriptomic evidence further implicates *PLIN5* and *PIK3R3* in the triceps brachii, genes known to regulate lipid metabolism and fatty acid composition [38,39], suggesting a potential molecular basis for flavor variation across muscle regions.

Integrated transcriptomic and non-targeted metabolomic analyses revealed a significant inverse relationship between *FBXO32* gene expression and carnosine content, while *GADL1* expression showed a positive correlation with carnosine levels. *FBXO32* encodes an E3 ubiquitin ligase, which functions as a biomarker for muscle atrophy and the deterioration of meat quality [40,41]. Carnosine has been shown by Rahman et al. [37] to inhibit skeletal muscle atrophy by suppressing the expression of muscle-specific E3 ubiquitin ligases, Atrogin-1/MAFbx and MuRF-1, thereby preventing the activation of the ubiquitin–proteasome system. In this study, elevated *FBXO32* expression in the HZ-T group, along with reduced carnosine levels relative to the HZ-B group, suggests that carnosine analogs enriched in the HZ-B group may inhibit the initiation of muscle atrophy, thereby improving meat quality. Significant enrichment of histidine and β-alanine metabolic pathways was revealed by KEGG co-enrichment analysis. *GADL1*, which functions as the rate-limiting enzyme in β-alanine synthesis, was found to have expression levels that directly regulate the efficiency of carnosine precursor supply [42]. A positive correlation between *GADL1* and carnosine was confirmed in this study, further validating the pivotal role of this enzyme in carnosine synthesis. Conversely, the significant downregulation of *FBXO32*, a recognized marker of muscle atrophy, suggests a potential role for carnosine in promoting muscle structural integrity. Based on its well-documented biological functions [43,44], we hypothesize a dual regulatory mechanism whereby carnosine may inhibit proteolytic pathways while simultaneously stabilizing structural proteins, thereby potentially contributing to the observed reduction in *FBXO32* expression and the enhancement of structural integrity. This proposed mechanism warrants further experimental validation.

This study provides new insights into the metabolic and molecular mechanisms underlying muscle heterogeneity in Hanzhong sheep. Nonetheless, certain limitations must be acknowledged. First, the flavor implications inferred from metabolite abundance were not validated by sensory evaluation or electronic tongue analysis. Given the complexity of flavor formation and its modulation by cooking, direct validation in future studies is necessary. Second, as the animals raised under free-range conditions, environmental variables such as diet seasonality and exercise may have influenced the metabolic profile. While this reflects realistic farming practices, controlled trials with standardized feeding and rearing systems will be essential to disentangle genetic versus environmental contributions. Addressing these limitations will strengthen the translation of metabolomic–transcriptomic insights into practical breeding strategies for improving meat quality.

## 5. Conclusions

Distinct metabolite profiles underlie the superior meat quality, tenderness, and low odor intensity of Hanzhong sheep. The longissimus dorsi was enriched in 1-methylhistidine, carnosine, anserine, and glucose metabolism intermediates, whereas the triceps brachii contained higher levels of acylcarnitines, L-glutamine, L-glutamate, inosinic acid, and taurine. These differences were closely linked to tissue-specific gene expression, including *GADL1*, *MYH2*, *MYBPC1*, *MYBPH*, *TPM1*, and *FBXO32*. Cross-breeding Hanzhong ewes with Suffolk or Nanqiu rams may enhance growth performance while preserving key traits—tenderness, flavor, and low odor—supporting the development of new meat sheep breeds adapted to the Qinba Mountain ecosystem.

## Data Availability

The original contributions presented in the study are included in the article, further inquiries can be directed to the corresponding authors.

## Abbreviations

TPA, texture-profile analysis; QC, Quality control sample; UHPLC-Q, Ultra-High Performance Liquid Chromatography–Quadrupole; HMDB, Human Metabolome Database; PCA, Principal Component Analysis; OPLS-DA, Orthogonal Partial Least Squares Discriminant Analysis; DEG, Differentially expressed gene; DM, Differential metabolites; MYBPH, myosin-binding protein H; TPM1, tropomyosin 1; MYBPC1, myosin-binding protein C1; MYH2, myosin heavy chain 2; IGFN1, immunoglobulin-like and fibronectin type III domain containing 1; MYOM3, myomesin 3; GADL1, glutamate decarboxylase-like 1; FBXO32, F-box protein 32; TBX15, T-box transcription factor 15.

## Appendix A

**Table A1:** The primer information involved in RT-qPCR.

Gene Symbol	Gene Name	Forward Primer (5′ to 3′)	Reverse Primer (5′ to 3′)	Amplicon Length (bp)	Annealing Temp (°C)
GAPDH	glyceraldehyde-3-phosphate dehydrogenase	ACTGGCAAAGTGGACATCGT	TTCCCGTTCTCTGCCTTGAC	124	58
MYBPH	myosin-binding protein H	GGAACCTGGCTGTAGGTGAC	CGATGGTCTCCTGGATGTGG	110	58
TPM1	tropomyosin 1	GGCATGAAAGTCATTGAGAGCC	CTTCTGAGAGCTCAGCCCG	186	60
MYBPC1	myosin-binding protein C1	AAAAGCCTCAAGGCGGAAGT	TCGAATGTGTACACCCGGC	191	58
MYH2	myosin-binding protein H	AAAAGCGCCAGGAGGC	TTTGCCAGATTCCTCCTTCTT	198	57
CSRNP1	cysteine- and serine-rich nuclear protein 1	ATGCCAGCTGCTTCCTCAAT	GCAAGAGGACAAGCTGAGGT	124	59
LMOD1	leiomodin 1	AAGGTAGCCAAGTATCGCCG	CTCCAGCTCCTCCATCTCCT	93	59
TBX15	T-box transcription factor 15	GCTCAGCCCTCTGAAACCTT	TTGCCTGCCTGCATGACATA	160	59

**Table A2:** Transcriptome quality control data. sample: sample name; raw reads: the total number of entries in the original sequencing data; clean bases: total sequencing data volume after quality control; total mapped: the number of clean reads that can be located on the genome; error rate: the average error rate of sequencing bases corresponding to the quality control data; Q20, Q30: Q20 and Q30, respectively, refer to the percentage of bases with sequencing quality above 99% and 99.9% among the total bases; GC content (%): the percentage of the total G and C bases corresponding to the quality control data in the total number of bases.

Sample	Raw Reads	Clean Reads	Total Mapped	Error Rate (%)	Q20 (%)	Q30 (%)	GC Content (%)
HZ-B1	50637628	50280308	49297209 (98.04%)	0.0122	98.65	95.6	51.38
HZ-B2	50451316	50076994	48893073 (97.64%)	0.0121	98.69	95.71	51.81
HZ-B3	48301048	47920416	46882259 (97.83%)	0.0122	98.66	95.62	52.65
HZ-T1	50634370	50266244	49270916 (98.02%)	0.0123	98.6	95.44	51.67
HZ-T2	49530508	49215030	48152042 (97.84%)	0.0122	98.64	95.57	53.44
HZ-T3	46321530	45951396	44966118 (97.86%)	0.0123	98.62	95.51	52.05
